# A Distinct Clinical Entity of Invasive Cardiac Aspergillosis: Not the Heart Valves This Time

**DOI:** 10.3390/jof11070486

**Published:** 2025-06-26

**Authors:** Zaid Al Khouri, Hunter Smeltzer, Anood Al Qura’an, Mohammad H. Khan, Alexandre E. Malek

**Affiliations:** Department of Medicine, Division of Infectious Diseases, Louisiana State University Health Sciences Center, Shreveport, LA 71103, USA; zaid.alkhouri@lsuhs.edu (Z.A.K.); hunter.smeltzer@lsuhs.edu (H.S.); anood.alquraan@lsuhs.edu (A.A.Q.); muhammad.khan03@lsuhs.edu (M.H.K.)

**Keywords:** *Aspergillus*, cardiac, pericardium, myocardium, invasive fungal infection

## Abstract

Invasive aspergillosis (IA) is a life-threatening infection that mainly affects immunocompromised hosts. Cardiac involvement is rare but can be the sole presentation of IA. It is associated with a high mortality rate and mostly occurs in patients with pre-existing cardiac disease. It can also be seen in immunocompetent patients with a structurally normal heart. The reported cases of cardiac involvement are usually due to infectious endocarditis (IE) caused by *Aspergillus* species (most commonly *Aspergillus fumigatus*). However, there is limited data on non-valvular cardiac aspergillosis (NVCA). We reviewed 67 cases of NVCA published between 1950–2024 and reported an additional case from our institution involving a 48-year-old female with kyphoscoliosis diagnosed with *Aspergillus* pericarditis.

## 1. Introduction

Aspergillosis is an infection caused by the ubiquitous *Aspergillus* species, primarily leading to invasive pulmonary infections in immunocompromised hosts [1]. Extrapulmonary manifestations, including cardiac involvement, are increasingly recognized in the medical literature and are associated with high morbidity and mortality rate [2,3]. Cardiac aspergillosis is often associated with worrisome outcomes, making it imperative for clinicians to have a thorough understanding of its various aspects [3]. Most of the reported cases of cardiac fungal infection describe valvular *Aspergillus* infective endocarditis as part of isolated or disseminated fungal infections [4].

However, non-valvular cardiac aspergillosis (NVCA) is a distinct and underrecognized clinical entity. Due to its rarity, nonspecific presentation, and absence of standardized diagnostic modalities, NVCA poses significant diagnostic and therapeutic challenges to clinicians. The literature on NVCA is scarce with only a few cases systematically analyzed [5]. In this current paper, we aimed through a systematic review to describe the clinical spectrum and burden of NVCA cases that are reported in the medical literature, and we added a case of isolated *Aspergillus* pericarditis in an immunocompetent patient seen at our institution.

## 2. Materials and Methods

**Figure 1:**
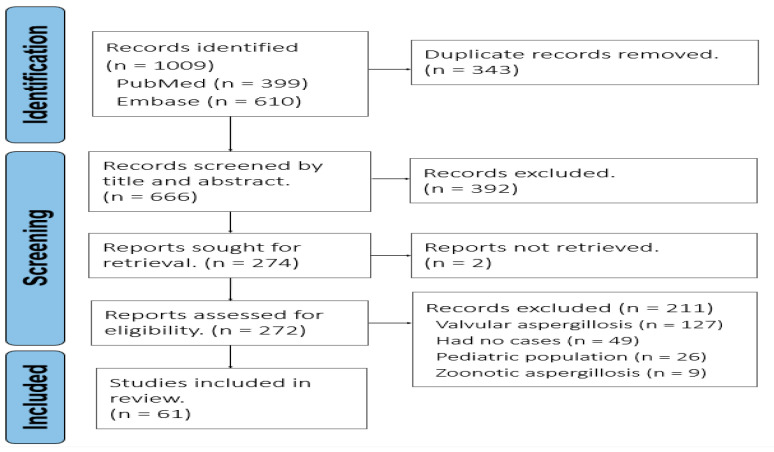
PRISMA flow diagram of study selection process.

The current study is a systematic review of all published case reports and series, supplemented by one clinical case seen at our institution. We reviewed all adult cases of NVCA reported between the year of 1950 and December 2024. Searches were performed in PubMed (*n* = 399) and Embase (*n* = 610), yielding a total of 1009 records (Figure 1). After removing 343 duplicates, 666 unique articles were screened by titles and abstracts. Of these, 274 full-text articles were assessed for eligibility, while two reports were unavailable for retrieval. Ultimately, 272 studies were reviewed in detail by two independent reviewers. The inclusion criteria were as follows: (1) adult patients (≥18 years), and (2) confirmed NVCA based on histopathological or microbiological evidence—this included fungal cultures, tissue cultures, or aerobic cultures from any cardiac tissue or pericardial fluid, with or without evidence of disseminated disease. The reports were excluded if the fungal infection involved cardiac valves exclusively, pediatric patients, or zoonotic infections. No language restrictions were applied. The study design was retrospective, utilizing published case reports and case series. All data were derived from retrospective literature sources. The data from the literature represent biological replicates of individual patients, with no technical replicates included. One additional case from our institution was included in the analysis. Each case corresponded to a unique patient. We did not analyze repeated tests or measurements from the same person. The data extracted included demographics, underlying comorbidities, clinical features, diagnostic methods (including laboratory and imaging modalities), organ involvement, antifungal treatments, procedural interventions, and patient outcomes. Diagnostic laboratory methods included histopathological examination with specific fungal staining, fungal cultures, and routine aerobic and anaerobic cultures from cardiac tissue or from pericardial fluid. Serum and fluid biomarkers, such as galactomannan antigen and (1–3)-β-D-glucan assays, were regarded as supportive rather than confirmatory diagnosis for invasive aspergillosis—galactomannan testing is primarily recommended in high-risk populations, such as patients with hematologic malignancies or those undergoing hematopoietic stem cell transplantation (HSCT), while (1–3)-β-D-glucan is recommended more broadly but lacks specificity for *Aspergillus*. The positive diagnostic criteria were standardized as either histopathological demonstration of hyphae consistent with *Aspergillus* or positive cultures from cardiac tissue or pericardial fluid. Biomarker positivity was defined using established cutoffs (e.g., galactomannan index ≥ 0.5) but always interpreted in conjunction with clinical, microbiological, and imaging findings.

## 3. Clinical Vignette

A 48-year-old female with a history of chronic hypoxic and hypercapnic respiratory failure due to severe congenital kyphoscoliosis was admitted with a chief complaint of worsening shortness of breath with hypoxemia (SpO_2_ 88% on room air) while remaining hemodynamically stable. Laboratory workup revealed a new diagnosis of diabetes mellitus (DM). The computed tomography (CT) of the chest revealed severe dextroconvex thoracic scoliosis, minimal atelectasis in the right upper lobe and in both lower lobes, small right pleural effusion and moderate pericardial effusion. There was radiological evidence of prior granulomatous disease, but there were no pulmonary cavities or consolidation. The electrocardiogram (ECG) described sinus tachycardia with nonspecific ST changes suggestive of a possible anterior infarct. The transthoracic echocardiogram (TTE) showed moderate to large pericardial effusion with fibrinous strands within the pericardial space consistent with early tamponade.

Accordingly, the patient underwent thoracotomy with pericardial window. The aerobic culture of pericardial fluid showed fungal growth at day 3 of incubation, but a sufficient quantity was not achieved until day 6 with preliminary identification *Aspergillus species*. The cultures were incubated on Mycosel and Sabouraud Dextrose Agar with Brain Heart Infusion (SABHI) slants at 29.4 °C. The isolate was sent to referent laboratory with identification finalized as *Aspergillus fumigatus* via Matrix-Assisted Laser Desorption/Ionization Time-of-Flight Mass Spectrometry. Antifungal susceptibility testing was performed using Clinical and Laboratory Standards Institute (CLSI) M38-A2 microdilution broth methodology, demonstrating minimum inhibitory concentrations (MICs) of 0.5 µg/mL for voriconazole, 0.5 µg/mL for amphotericin B, and >64 µg/mL for fluconazole. The histopathological examination showed no obvious granulomas or fungal elements on Gomori methenamine silver. Pathology sections showed mesothelial lining fibrous tissue and hyperplasia. No malignant cells were noted. The Acid-Fast Bacilli (AFB) and fungal cultures for other organisms were negative. Serum *Aspergillus* antigen (galactomannan) performed using the FDA-cleared Bio-Rad Platelia *Aspergillus* Galactomannan EIA by Mayo Clinic Laboratories, Fungitell Assay For (1–3)-B-D-Glucans performed using the FDA-cleared Fungitell Assay, a kinetic ELISA based on modification of the Limulus Amebocyte Lystate pathway by Mayo Clinic Laboratories, and *Aspergillus* spp. The *Aspergillus* antigen and PCR testing on pericardial fluid were not performed. The remaining serum endemic mycoses workup, HIV testing, T-Spot, and rapid plasma reagin (RPR) were all negative including fungal immunodiffusion antibodies testing. Infectious diseases were consulted and initiated intravenous (IV) liposomal amphotericin B (L-AmB) 3 mg/kg daily for 5 days, followed by oral voriconazole 4 mg/kg twice daily (maintenance dose). Given the persistence of output from the pericardial drain, it was replaced with a PleurX^®^ catheter, which later became dislodged and was subsequently removed after 10 days.

Given the unusual case of *Aspergillus* infection involving only the pericardium in the absence of a compromised immune system, a workup was performed to rule out primary adult-onset immunodeficiency. The workup revealed mild eosinophilia with a count of 0.536 K/uL and elevated total IgE (624 IU/mL) which might have been secondary to *Aspergillus* infection. She was discharged to a rehabilitation facility on oral voriconazole 200 mg twice daily (BID). She was followed in the outpatient settings with in-depth immunological evaluation by Allergy and Immunology and Infectious Diseases specialists, including T-helper 17 (Th17) enumeration, which was within normal limits, and her IgE dropped to 228 IU/mL while on antifungal therapy; thus, she was less likely to have hyper IgE syndrome. She showed continued clinical improvement and required supplemental oxygen only with exertion. She completed a 6-month course of voriconazole with no major adverse events noted. Two months later, a repeat TTE revealed no residual pericardial effusion or structural abnormalities.

## 4. Results

We identified a total of 67 cases with NVCA, including the case we presented (Table 1 and Supplementary Table S1) [3,5,6,7,8,9,10,11,12,13,14,15,16,17,18,19,20,21,22,23,24,25,26,27,28,29,30,31,32,33,34,35,36,37,38,39,40,41,42,43,44,45,46,47,48,49,50,51,52,53,54,55,56,57,58,59,60,61]. The study included 24 female (36%) and 43 male patients (64%). The mean age was 44.6 years ranging from 18–79 years old. We noted 10 patients (15%) with no underlying medical conditions, but 3 had potential risk factors, including a patient with smoking history, and the other two with a potentially hazardous occupation (one was a diamond cutter, and the other did labor in shipyard, chemical factory, bricklayer apprentice, peanut factory). Our patient had no significant medical or surgical history other than chronic respiratory failure, advanced congenital kyphoscoliosis, and newly diagnosed diabetes mellitus. The remaining 56 patients (83.5%) had an underlying medical or surgical history with immunodeficiency, as 20 patients had hematologic malignancy (30%) including five patients with acute myeloid leukemia (AML, 7.5%), 4 patients with acute lymphoblastic leukemia (ALL, 6%), 3 patients with chronic myeloid leukemia (CML, 4.5%), 3 patients with chronic lymphocytic leukemia (CLL, 4.5%), 4 patients with lymphoma (6%), whether mantle cell, lymphoblastic, or Hodgkin’s, and 1 patient with myelodysplastic syndrome (MDS, 1.5%). There were four other patients with underlying hematologic disease (6%), including aplastic anemia, refractory anemia, disseminated intravascular coagulation (DIC), and neutropenia status post splenectomy. We identified 16 patients who had transplant history (24%), including 8 with hematologic stem cell transplantation (HSCT, 12%), 3 patients with renal transplant (4.5%), 3 patients with liver transplant (4.5%), and 1 patient with heart and another patient with lung transplant. Acquired or congenital immunodeficiency with the use of immunosuppressive agents including steroids was noted in 5 patients (7%) who had hypogammaglobulinemia, chronic granulomatous diseases, and hemophagocytic syndrome, and 4 patients had human immunodeficiency virus/acquired immunodeficiency syndrome (HIV/AIDS, 6%). Also, 5 patients had DM (7%). The remaining underlying medical illness included 4 patients with hypertension (HTN) (6%), 1 patient with hyperlipidemia (HLD) (1.5%), 6 with underlying renal conditions (9%), 3 with gastrointestinal disorders (4.5%), 5 with hepatobiliary disease (7%), 2 with spine disorders (3%), 10 with rheumatologic disorders (15%), 9 patients with respiratory conditions (13.5%), and 9 patients with cardiac disorders (13.5%).

The most common clinical manifestation was fever, reported in 33 patients (49%). The respiratory symptoms were frequent, including shortness of breath ranging from mild dyspnea to respiratory failure in 31 patients (46%), cough in 14 patients (21%), of whom 6 (9%) had hemoptysis, and chest pain in 10 patients (15%). Gastrointestinal symptoms were present in 14 patients (21%), with abdominal pain in 8 patients (12%) and nausea, vomiting, and/or diarrhea in 6 patients (9%). Neurological or ocular manifestations, including altered mental status, were reported in 10 patients (15%). Hemodynamic instability was observed in 15 patients (22%) who were presented with hypotension or shock. End-organ damage was documented in 11 patients (17.5%), including cardiac arrest (*n* = 3), renal impairment (*n* = 3), liver impairment (*n* = 4), and one case with unspecified multi-organ failure.

The diagnosis of NVCA was established via cultures and/or histopathology either from the pericardial fluid, cardiac masses, coronaries, or from the cardiac tissue layers. The diagnosis was made antemortem in only 27 cases (40%), 38 cases (57%) were diagnosed postmortem, and in 2 cases (3%) were diagnosed antemortem and confirmed postmortem. Cardiac involvement was most commonly limited to the pericardium (39%), followed by pancardial involvement (27%), and myocardium alone (15%) and other layers involvement as listed in Table 1. The lung involvement was noted in 45 patients (67%), while disseminated aspergillosis—with or without pulmonary involvement—was documented in 35 patients (52%). The laboratory diagnostic data were available for only 19 patients (28%), with 13 showing positive results (19%) and 6 showing negative results (9%). Positive findings were reported in serum, bronchoalveolar lavage (BAL), or pleural fluid, including detection of (1–3)-β-D-glucans, galactomannan, or *Aspergillus* antibodies. In one case, *Aspergillus* DNA was detected retrospectively in a postmortem serum sample. Although most patients had a TTE, findings were documented in only 40 cases (60%). Among these, two had normal results, while pericardial effusion or tamponade was reported in 31.5%. Other echocardiographic findings included reduced ejection fraction, cardiac masses, valvular vegetations, fibrinous deposits/strands, and pneumopericardium. *Aspergillus* species was identified in all patients. The species of *Aspergillus* was unspecified in 49%, whereas species identification was reported in 34 patients (51%). The most frequently isolated species was *Aspergillus fumigatus* (23 cases, 34%), followed by *A. flavus* (12%), *A. nidulans* (3%), and *A. niger* (2%). Co-infection with other pathogens was reported in 29 cases (43%), whereas 38 cases (57%) had no documented co-infection (Table 1 and Supplementary File S1 for variable definitions).

Of the 67 patients with NVCA, treatment strategies varied widely (Table 2 and Supplementary Table S2). The most common approach was antifungal treatment as monotherapy (*n* = 14), including 11 patients who received L-AmB, all of whom died. A combination of antifungal therapy and surgical or procedural intervention was adopted in eight patients, with a survival rate of 37.5% (3/8). Combination of antifungal therapy without surgical intervention was considered in six cases, with two survivors (33%). The sequential antifungal therapy with surgical approach (*n* = 4) was associated with three survivors (75%), while sequential therapy alone in two patients with one patient who died. Antifungal treatment as monotherapy combined with surgery or procedures was noted in 11 cases, with 5 survivors (45.5%), including 3 who received voriconazole; the remaining 6 patients, all of whom died, had received L-AmB. Three patients received surgery or invasive procedures only, with two documented deaths and one case with an undocumented outcome. Notably, 19 patients received no antifungal or surgical treatment; all of these cases were fatal, except for 2 with no documented outcome.

Amphotericin B (AMB) was the most used antifungal agent, administered in 30 patients (45%) either alone or in combination with other antifungals. Of these, 6 (20%) survived and 24 patients (80%) died. L-AmB was used in five cases (7.5%), with two survivors and three deaths, including one patient who died despite also receiving it intrapericardially. Dosages ranged from 1 to 5 mg/kg/day, with treatment durations between 2 and 93 days. Amphotericin B deoxycholate (ABD) was administered in 11 patients (16.5%), with a survival rate of 18% (2/11). Dosing schedules varied and were often unspecified. In 13 cases (19.5%), the specific formulation of AMB was undocumented. Of these, 2 patients (15%) survived and 11 died, including one who had also received intrapericardial AMB.

Azole antifungals were administered to 21 patients (31%), either as monotherapy or in combination with other antifungals, and were associated with a more favorable survival profile. Overall, 12 of 21 patients (57%) survived, including 9 of 15 who received voriconazole. The most frequently used azole was voriconazole (22%). Although dosing regimens were undocumented in most cases, those reported involved a loading dose of 6 mg/kg twice daily on day 1, followed by 4 mg/kg twice daily for 1 to 6 months. Fluconazole was administered to four patients (6%), typically before a confirmed diagnosis of NVCA, and was associated with 100% mortality. Itraconazole was used in three patients (4.5%), always in combination with other agents. The echinocandins were used in nine patients (13.5%), always in combination with other antifungal agents, with a survival rate of 44.4% (4 of 9). Caspofungin was the most common used echinocandin (10.5%) and was given to seven patients, with three survivors. In most of these cases, dosing involved 70 mg on day 1 followed by 50 mg daily for duration ranging from 3 to 7 weeks. Micafungin and anidulafungin were each used in one patient. The patient who received micafungin died, while the one treated with anidulafungin survived. Dosing schedules and treatment durations were inconsistently reported across cases. Flucytosine (5-FC) was used in three patients (4.5%) along other antifungal agents, and all patients died.

Procedural interventions were performed in 27 of 67 patients (40%), either as single or multiple procedures, with or without concurrent antifungal therapy. Among these patients, 11 (41%) survived, 15 (56%) died, and the outcome was unknown in 1 case. Pericardiocentesis was performed in nine patients (13.5%), with a survival rate of 44% (4/9). Pericardial drainage or window creation was performed in 10 patients (15%), with a higher mortality rate (70%, 7/10). Cardiac mass excision was performed in five patients with two patients surviving. Pericardiectomy, with or without pleuropericardial fenestration, was performed in four cases, achieving a 75% survival rate (3/4). Other procedures included pericardial fibropurulent tissue removal and percutaneous transluminal coronary angioplasty.

## 5. Discussion

NVCA remains a rare and underrecognized manifestation of invasive aspergillosis (IA), typically overshadowed by the more commonly reported valvular infective endocarditis [62]. Our systematic review highlights that NVCA predominantly affects immunocompromised patients [63]—particularly those with hematologic malignancies or solid organ transplants—but it can also occur in immunocompetent individuals. The cardiac involvement can be the sole presentation of IA [10]. This clinical entity coupled with nonspecific presentation contributes to frequent delays in establishing the diagnosis. Most cases were diagnosed postmortem [48,50], and in many instances, no fungal-specific laboratory testing was performed during hospitalization [7,8]. These findings emphasized a major diagnostic gap and the unmet need for heightened clinical suspicion when encountering unexplained cardiac or pericardial pathology.

Our review demonstrates that prompt recognition and early initiation of antifungal therapy significantly impact on patient outcomes. Voriconazole alone or in combination with other antifungal therapy, including L-AmB, was associated with the most favorable survival rates. Procedural/surgical interventions such as pericardial drainage or pericardiectomy were frequently adopted and offered additional therapeutic benefit when combined with antifungal treatment. However, inconsistencies in antifungal regimens, formulations, route of administration, and treatment durations across cases highlight the need for standardized therapeutic guidelines. These survival discrepancies reinforce the importance of treatment standardization and suggest that regimens incorporating early combination or sequential antifungal therapy and timely procedural intervention may offer the best outcomes. NVCA has been described as affecting any or all cardiac layers—either in isolation or in combination. The clinical features may include pericarditis, myocarditis, pancarditis, coronary artery embolization, or cardiac masses [6,10,15,33,34]. These diverse clinical manifestations further complicate the diagnosis, especially in patients without known predisposing risk factors. Furthermore, species-level identification of *Aspergillus* was achieved in only half of the cases, underscoring the importance of comprehensive mycological evaluation for both diagnosis and treatment planning [53,56].

Our analysis of 67 NVCA cases revealed stark differences in outcomes based on treatment strategies. Antifungal treatment as monotherapy—most commonly L-AmB—was associated with 100% mortality [26,28,30]. In contrast, combination antifungal therapy, particularly when coupled with surgical or procedural intervention, was associated with improved survival [11,20,37]. The highest survival rates were observed in patients receiving sequential antifungal therapy plus surgery (75%) [6,10], and those undergoing monotherapy plus surgery (45.5%) [8,9]. These findings support an aggressive multimodal treatment approach and challenge the adequacy of antifungal monotherapy alone, particularly with L-AmB.

The presented case illustrates the potential benefit of a timely diagnosis and targeted antifungal therapy, even in patients without classic risk factors. The patient’s elevated IgE level raised suspicion for an underlying immunologic disorder such as hyper-IgE syndrome (HIES), particularly given her congenital kyphoscoliosis [64,65]. While further workup was inconclusive for HIES, her declined IgE levels were in correlation with clinical improvement and treatment response in extrapulmonary aspergillosis. Although the trend of IgE level was traditionally studied in patients with allergic pulmonary aspergillosis (ABPA) [3], this association has not been well explored in cardiac aspergillosis. Future prospective studies are needed to evaluate whether IgE can aid in monitoring treatment response or predicting outcomes in NVCA.

The findings of this study have significant clinical implications. Clinicians should consider NVCA in the differential diagnosis of pericardial effusion or carditis, particularly in patients with hematologic malignancies, recent transplantation, or unexplained immunologic findings in a suggestive clinical context. Even immunocompetent patients presenting with unexplained pericardial disease may warrant evaluation for fungal pathogens. Non-invasive diagnostics, including serum galactomannan [66], (1–3)-β-D-glucan [67], and echocardiography, should be incorporated early in the workup [68,69]. In selected cases, total IgE levels may offer additional insights, though further validation is needed. Establishing a registry of NVCA cases and encouraging standardized reporting of antifungal susceptibility, antifungal treatment duration, and outcomes would help refine clinical guidelines and improve recognition of this potentially fatal fungal infection.

This review has several limitations. It is based on retrospective case reports and series, which are inherently subject to selection bias, reporting inconsistencies, and incomplete data. Many studies lacked full diagnostic or therapeutic details, and clinical outcomes were sometimes unspecified. The rarity of NVCA and the predominance of autopsy-confirmed cases likely skew the data toward more severe presentations, potentially underestimating the true spectrum of disease. Moreover, the variability in antifungal strategies and lack of consistent susceptibility data limit our ability to draw definitive conclusions about optimal therapy and duration. Lastly, the case we presented, while illustrative, may not be generalizable due to its unique clinical context.

## 6. Conclusions

NVCA remains rare and underrecognized, but a highly fatal clinical entity. Clinicians should maintain a high index of suspicion, particularly in patients with liquid tumors and pericardial effusion. Our findings also highlight on the importance of considering NVCA in immunocompetent individuals when the clinical context is suggestive. If NVCA is suspected, initial evaluation includes reasonable non-invasive diagnostic measures such as serum (1–3)-B-D-Glucans, galactomannan assays, and TTE. The role of total serum IgE as a biomarker in extrapulmonary *Aspergillus* infection and its potential utility in monitoring treatment response warrant further investigation. A combined approach involving antifungal therapy, particularly voriconazole—alongside minimally invasive or surgical intervention—was associated with favorable outcomes in most cases. Given the high mortality, an early initiation of antifungal therapy and prompt consideration of surgical intervention should be prioritized whenever feasible.

## Figures and Tables

**Table 1:** Baseline characteristics of all patients with NVCA.

Variables	Total (*n* = 67)
**Demographics**	
Age (mean, range)	44.6 years (18–79)
Male sex	43 (64%)
**Comorbidities**	
Hematologic Malignancies (Leukemia, Lymphoma, MDS)	20 (30%)
Other Hematologic disorders	4 (6%)
Transplant history	16 (24%)
HIV/AIDS	4 (6%)
Primary Immunodeficiency	3 (4.5%)
Chronic steroids	2 (3%)
Diabetes mellitus	5 (7.5%)
Hypertension	4 (6%)
Cardiac disorders	9 (13.5%)
Pulmonary disorders	9 (13.5%)
Gastrointestinal disorders	3 (4.5%)
Hepatobiliary disorders	5 (7.5%)
Rheumatologic disorders	10 (15%)
Spine disorders	2 (3%)
**Clinical presentation**	
Fever	33 (49%)
Shortness of breath	31 (46%)
Cough (±hemoptysis)	14 (21%) (±6 [9%])
Chest pain	10 (15%)
Abdominal pain	8 (12%)
Nausea, vomiting, and/or Diarrhea	6 (9%)
Neurologic/Ocular signs	10 (15%)
Skin manifestations	3 (4.5%)
Shock or hypotension	15 (22%)
Other end-organ damage	11 (17.5%)
**Echocardiogram findings**	
Pericardial effusion/Tamponade	33 (49%)
Cardiac mass	23 (34%)
Valvular vegetation	8 (12%)
Fibrin deposits/bridges	2 (3%)
Pneumopericardium	1 (2%)
Low ejection fraction	33 (49%)
**Level of cardiac involvement**	
Pancardium	18 (27%)
Pericardium alone	26 (39%)
Myocardium alone	10 (15%)
Myocardium and Endocardium	5 (7%)
Pericardium and Endocardium	2 (3%)
Pericardium and Myocardium	1 (2%)
Cardiac mass	5 (7%)
**Systems involved in Invasive Aspergillosis**	
NVCA alone	14 (21%)
NVCA + Lung	10 (15%)
NVCA + Lung + Other distant organs	35 (52%)
***Aspergillus* species Identified**	
Not specified	33 (49%)
*A. fumigatus*	23 (34%)
*A. flavus*	8 (12%)
*A. nidulans*	2 (3%)
*A. niger*	1 (2%)
**Concomitant infections**	
Gram negative bacteremia	8 (12%)
Gram positive bacteremia	16 (24%)
Viral (unspecified)	9 (13%)
Respiratory coinfections	15 (22%)
Cardiac coinfections	4 (6%)
Gastrointestinal infections	9 (13%)
Wound infections	2 (3%)
Leptospirosis	4 (6%)

**Table 2:** NVCA patient outcome according to treatment status.

	Total Patients ^1^ (*n*, %)	Survived (*n*, %)	Died (*n*, %)
**Untreated ^2^**	**19 (28%)**	**0 (0%)**	**17 (89.5%)**
**Amphotericin B (AMB)**	**30 (45%)**	**6 (20%)**	**24 (80%)**
IV L-AmB +/− IP ^3^	5 (7.5%)	2 (40%)	3 (60%)
IV ABD	11 (16.5%)	2 (18%)	9 (82%)
IV ABD switched to IV LAmB	1 (1.5%)	0 (0%)	1 (100%)
IV AMB Not specified ± IP ^4^	**13 (19.5%)**	**2 (15%)**	**11 (85%)**
**Azole ^5^**	**21 (31%)**	**12 (57%)**	**9 (43%)**
Voriconazole	15 (22%)	9 (60%)	6 (40%)
Fluconazole	4 (6%)	0 (0%)	4 (100%)
Itraconazole	3 (4.5%)	1 (33%)	2 (67%)
Isavuconazole	1 (1.5%)	1 (100%)	0 (0%)
Ketoconazole	1 (1.5%)	1 (100%)	0 (0%)
**Echinocandin**	**9 (13.5%)**	**4 (44%)**	**5 (56%)**
Caspofungin	7 (10.5%)	3 (43%)	4 (57%)
Micafungin	1 (1.5%)	0 (0%)	1 (100%)
Anidulafugin	1 (1.5%)	1 (100%)	0 (0%)
**Flucytosine**	**3 (4.5%)**	**0 (0%)**	**3 (100%)**
**Antifungal—Not specified**	**4 (6%)**	**2 (50%)**	**2 (50%)**
**Procedure ^6^**	**27 (40%)**	**11 (40.7%)**	**15 (55.6%)**
Pericardiocentesis	9 (13.5%)	4 (44.5%)	5 (55.5%)
Pericardial drain or window	10 (15%)	3 (30%)	7 (70%)
Mass excision ^7^	5 (7.5%)	2 (40%)	2 (40%)
Valve replacement	3 (4.5%)	1 (33%)	2 (67%)
Pericardial purulent tissue removal	1 (1.5%)	1 (100%)	0 (0%)
PTCA ^8^ attempted	1 (1.5%)	0 (0%)	1 (100%)

^1^ Patients have received either of those treatment groups alone or in different combinations in the same group or with different groups. ^2^ Two patients out of 19 who were not treated, the outcome was not documented. ^3^ One patient who received LAmB also received intrapericardial amphotericin B; this patient died. ^4^ One patient who received non-specified amphotericin B also received intrapericardial amphotericin B; this patient died. ^5^ Some patients in this group alternated between different azoles in the same hospital course. ^6^ Some patients in this group underwent multiple procedures in the same hospital course. ^7^ One patient who underwent cardiac mass excision is outcome unknown. ^8^ Percutaneous transluminal coronary angioplasty.

## Supplementary Materials

The following supporting information can be downloaded at: https://www.mdpi.com/article/10.3390/jof11070486/s1, Table S1: Legend” Comprehensive details of all cases reported in the medical literature”; Table S2: Legend “Comprehensive details about the reported treatment strategies”; File S1 Legend “Supplementary Materials for Baseline Characteristics of NVCA Patients”.
