# Supplementary material for: A Distinct Clinical Entity of Invasive Cardiac Aspergillosis: Not the Heart Valves This Time
Source: J Fungi (Basel). 2025 Jun 26;11(7):486. doi: 10.3390/jof11070486 (PMC12295503; doi:10.3390/jof11070486)
Supplement: Supplementary file 1 [file jof-11-00486-s001.zip › Supplementary File S1.pdf]

Supplementary Materials for Baseline Characteristics of NVCA Patients

1. Comorbidities

- Hematologic malignancies (n=20, 30%): Includes AML (5), ALL (4), CML (3), CML (3), lymphoma (4), and MDS (1).
- Other hematologic disorders (n=4, 6%): Includes aplastic anemia (1), refractory anemia (1), DIC (1), and splenectomy 2/2 neutropenia (1).
- Transplant history (n=16, 24%): Includes stem cell or bone marrow (8), solid organ including renal (3), liver (3), heart (1), and lung (1).
- HIV/AIDS (n=4, 6%)
- Primary immunodeficiency (n=3, 4.5%): Includes pan hypogammaglobulinemia (1), hemophagocytic syndrome (1), and chronic granulomatous disease.
- Chronic steroid use (n=2, 3%)
- Diabetes mellitus (n=5, 7.5%)
- Hypertension (n=4, 6%)
- Cardiac disorders (n=9, 13.5%): Includes valvular (2), coronaries(2), cardiomyopathies (2), heart failure (1), arrhythmia (1), & bacterial pericarditis (1)
- Pulmonary disorders (n=9, 13.5%): Includes Asthma/COPD (4), TB +/- ATT (3), cystic fibrosis (1) and a case of lung cancer (1).
- GI disorders (n=3, 4.5%): Includes peptic ulcer disease (1) and inflammatory bowel disease (2).
- Hepatobiliary disorders (n=5, 7.5%): Includes a case each of cirrhosis from HBV, HCV, & 1ry biliary cirrhosis, halothane induced liver failure, & an unspecified case.
- Rheumatologic disorders (n=10, 15%): Includes 2 SLE, and a case each of IgA vasculitis, leukocytoclastic vasculitis, Rheumatic heart disease, Antiphospholipid, Sjogren's, Churg Straus, Sarcoidosis, & polyarthritis.
- Spine disorders (n=2, 3%): Includes a case of cervical disc disease and Kyphoscoliosis.

2. Clinical Presentation

- Fever (n=33, 49%)
- Shortness of breath (n=31, 46%)
- Cough (n=14, 21%), including hemoptysis (n=6, 9%)
- Chest pain (n=10, 15%)
- Abdominal pain (n=8, 15%)
- Nausea, vomiting, and/or Diarrhea (n=6, 9%)
- Neurologic/ocular signs (n=10, 15%)
- Skin manifestations (n=3, 4.5%)
- Shock or hypotension (n=15, 22%)
- Other end-organ damage (n=11, 17.5%): Includes 4 liver failure, 3 renal failure, 3 cardiac arrests, and 1 not specified multi-organ failure

3. Echocardiographic Findings

- Pericardial effusion / tamponade (n=33, 49%)
- Cardiac mass (n=23, 34%)
- Valvular vegetation (n=8, 12%)
- Fibrin deposits / strands (n=2, 3%)
- Pneumopericardium (n=1, 2%)
- Low ejection fraction (n=33, 49%): Defined as <40% when specified.

4. Cardiac Involvement Definitions

- Pericardium alone (n=26, 39%)
- Pancardium (n=18, 27%)
- Myocardium alone (n=10, 15%)
- Myocardium + endocardium (n=5, 7%)
- Pericardium + endocardium (n=2, 3%)
- Pericardium + myocardium (n=1, 2%)
- Cardiac mass only (n=5, 7%)

5. Systemic Involvement

- NVCA alone (n=14, 21%)
- NVCA + lung only (n=10, 15%)
- NVCA + lung + other organs (n=35, 52%)

6. Species Identification

- Not specified (n=33, 49%)
- A. fumigatus (n=23, 34%)
- A. flavus (n=8, 12%)
- A. nidulans (n=2, 3%)
- A. niger (n=1, 2%)

7. Concomitant Infections

- Gram-negative bacteremia (n=8, 12%) includes 2 *Enterobacter spp.*, *2 E. coli*, a case each of *Salmonella, Brevundimonas, Kluyvera*, and 2 unspecified bacteria.
- Gram-positive bacteremia (n=16, 24%) includes 5 *Staph epidermidis*, 2 *Strep spp*. (*S. agalactiae & Strep. sanguinis*), 2 Enterococcus (*E. faecalis & VRE*), and 1 lactobacillus.
- Viral infections (n=9, 13%) includes 4 CMV, 2 EBV, 1 HSV, 1 adenovirus, and 1 Parvovirus B19.
- Respiratory coinfections (n=15, 22%) includes 4 Pseudomonas, 2 Enterobacter, 2 Enterococcus, 2 *Staph epidermidis*, 3 *Candida spp.*, 1 *Toxoplasma* & 1 *Pneumocystis pneumonia*.
- Cardiac coinfections (n=4, 6%) include 2 Enterococcus Infective Endocarditis, 1 Strep Agalactiae pericarditis, & 1 cardiac toxoplasmosis.

- GI infections (n=9, 13%) includes 3 Candida spp., 2 CDI, 1 HSV, 1 Giardia, 1 Kluyvera, and 1 salmonella.
- Wound infections (n=2, 3%) include a case of *Pseudomonas* and a case of *Staphylococcus epidermidis.*
- Leptospirosis (n=4, 6%)

Note: These definitions and case counts were applied consistently in the construction of Table 1 for descriptive consistency and to support comparability across the heterogeneous case reports and series analyzed in this study.
