# Supplementary material for: A Distinct Clinical Entity of Invasive Cardiac Aspergillosis: Not the Heart Valves This Time
Source: J Fungi (Basel). 2025 Jun 26;11(7):486. doi: 10.3390/jof11070486 (PMC12295503; doi:10.3390/jof11070486)
Supplement: Supplementary file 1 [file jof-11-00486-s001.zip › Supplementary Table S1.pdf]

Table S1. Comprehensive details of all cases reported in the medical literature.

Author, Year, type of article, & # of case	Age and Sex	Clinical picture	Medical History	Aspergil-lus spp.	Cardiac level	Lab	Radiological findings	TTE	Dx Ante Vs Post mortem	Extra cardiac	Treatment	Outcome
Our case, CR: 1	48 F	Myalgia, SOB with hypoxia	Scoliosis	fumigatus	PERI	Galactoman-nan, fungitell both negative in serum	Scoliosis, plural and pericardial effusion	Tamponade & fibrin deposit	AM	N.D.	Pericardial window 2 weeks + L-AmB 3 mg/kg/d IV then PO VOR 300 mg BID D1 then reduced to 200 mg BID for 6 months	Alive
(Walsh & Bulkley, 1982), CS: 2, 3, 4, & 5	29 F	Fever and low BP	Renal trans-plant	Species	PAN	N.D.	Wide-spread infiltrates	N.D.	PM	Lung, brain, liver, gut, spleen, thyroid.	No treatment	Died (N.D.) "Septic emboli."
	61 F	Fever and low BP	CLL	Flavus	PERI & MYO	N.D.	RLL infiltrate Wide cardiac diameter.	N.D.	PM	Lungs, larynx, kidneys, esoph-agus, stomach, vagina	ABD 0.6-1 mg/kg/day (Duration N.D.)	Died (N.D.) "pericardial tamponade."
	27 F	Fever, low BP & respiratory distress	CML	Flavus	PERI	N.D.	Plural effusion	Large pericardial effusion	PM	Lung	Pericardiocentesis + ABD 0.6-1 mg/kg/d & 5-FC (Dose/Duration N.D.)	Died (N.D.) "pericardial tamponade."
	29 F	Fever and SOB	AML & did BMT	Flavus	PAN	N.D.	LLL nodule/ infiltrate	N.D.	PM	Lung, brain, kidneys, esoph-agus, and breast	ABD 0.6-1 mg/kg/day (Duration N.D.)	Died (N.D.) "Pulmonary aspergillus"
(Le Moing et al., 1998), CR: 6	33 F	Fever post Chemo	DM, ALL	Species	PERI	+ve Aspergil-lus antigen in pericardial fluid	Round LUL, peri lesion halo; nodules & mild right pleuritis	Cardiac Tamponade	AM	Pleural fluid	ABD 1.5 mg/kg/d (To-tal 1.8 g) for 1 month followed by pericardi-ectomy, & pleuroperi-cardial fenestration and post-op switched to Itraconazole 200	Alive after aspergillosis but died a year later due to E.Coli shock

											mg TID for 1 week then BID for 6 months then QD 6 months then he died	
(Biso et al., 2017), CR: 7	61 M	Fluid Overload, AMS posttransplant with VRE septic emboli	HTN, Renal transplant, ALF 2/2 HCV	Fumigatus	PERI (+ VRE in MV)	N.D.	Multiple septic emboli in the brain	Cardiac Tamponade & MV veg	AM	N.D.	Pericardiocentesis with pericardial drain placement & AMB Started the day the patient died	Died within 30 days
(Alkuwaiti et al., 2019), CR: 8	36 F	Abdomen pain, SOB, IE s/p splenectomy & double valve replacement	Rheumatic heart, Anti phospholipid syndrome	Species	PERI (& Enterococcus faecalis *in MV & AV)*	N.D.	Bilateral exudative plural effusion; splenic abscess	Pericardial effusion with MV & AV veg	AM	Lung	Fibropurulent PERI tissue resection then VOR 6 mg/kg BID for D1 & 4 mg/kg BID for 1 week then 200 mg for 8 weeks at D/C.	Alive
(Guimaron et al., 2022), CR: 9	78 F	Inspirational epigastric pain	HTN, CLL, erosive arthritis, leukocytoclastic vasculitis	Fumigatus	PERI & MYO	Galactomannan +ve in serum then -ve after Tt.	Fibrinous pericardial deposit. PET +ve LAD in mediastinum, lung, & PERI	Cardiac tamponade & fibrin deposit	AM	Lung, muscle Lymph node, & Subcutaneous	Pericardiocentesis + VOR (Dose/duration N.D.) then had pericardiectomy	Alive
(Hayashi et al., 2017), CR: 10	30 M	Fever and cough	None	Species	PAN	High fungitell & +ve serum Galactomannan	Pericardial effusion & pericardial space postive nuclear scan	Pericardial effusion and MV veg	AM	N.D.	Pericardiocentesis + VOR 400 mg/day for 37 days switched to AMB 150 mg/daily then back to VOR on day 60 2/2 AKI & d/c on it at day 148	Alive

(Kupsky et al., 2016), CR: 11	18 M	Persistent fever and high HR post transplant	Orthotopic liver transplant	Fumigatus	PERI	N.D.	Pericardial effusion with 3-cm lateral LV wall mass	Pericardial effusion, fibrin & mass	AM	N.D.	Pericardiocentesis followed by VOR & Anidulafungin (Dose/duration N.D.)	Alive
(Dalhoff et al., 1996), CR: 12	42 M	Progressive dyspnea and temperature of 37.8 °C then hypotension.	HIV/AIDS + Salmonella sepsis, pneumococcal peritonitis, & appendicitis	Fumigatus	PERI	N.D.	Enlarged heart & BL pleural effusion.	Cardiac tamponade	AM	N.D.	Pericardial drain then D3 started LAmB 1mg/kg/day + 2 mg AMB in 5% glucose intrapericardially per day but died after 2 days	Died within 30 days
(Schwartz, 1989), CR: 13	34 M	Hemoptysis, left side chest pain 8 weeks posttransplant	CML s/p BMT c/b GVHD	Fumigatus	PAN	N.D.	Bilateral pulmonary infiltrates	N.D.	PM	N.D.	ABD (Dose/duration N.D.)	Died within 30 days
(Alam et al., 1998), CR: 14	21 M	Fever and transient dysarthria	Hodgkin's lymphoma S/P CHOP & BMT	Fumigatus	ENDO like mass	N.D.	Apical lung scarring & BL cerebellar infarct	Mass (2.5 cm) from left upper PV & LAA by TEE	AM	Lung	Surgical excision of the mass then AMB post-op (Dose/duration N.D.)	Died within 30 days
(Itoh et al., 2006), CR: 15	38 M	Pyrexia & cough	Aplastic anemia, left nephrectomy in childhood	Species	PERI, MYO, & Coronaries	Galactomannan & fungitell +ve in serum	LLL with a fungus ball	N.D.	PM	Lung and Brain	AMB 0.5 mg/kg/day for 3 weeks then up to 0.7 mg/kg/day for 3 more weeks + failed PTCA	Died within 90 days. "MI S/P failed PTCA"
(Luce et al., 1979), CR: 16	38 F	Fever, edema post CTx & toxic enteritis followed by candidiasis	AML s/p allogeneic BMT	Niger	PERI	N.D.	Small RML infiltrate with enlarged cardiac silhouette	Pericardial effusion	BOTH	Aorta, lung, pleurae, Heart (not specified), renal, intestines thyroid	ABD 25 mg/kg prophylaxis started prior to BMT for 750 mg total + Pericardial window	Died within 30 days "Massive hemoptysis and died of VFib".

(Mortensen et al., 2011), CR: 17	40 F	Symptoms of de-compensated pericardium (Not specified)	CGD, IBD s/p resection of the Ileum and colon	Fumigatus	PERI	Galactomannan -ve in Serum but high Aspergillus IgG AB titers	Increase in cardiac silhouette. Bi-apical pleural thickening, pericardial effusion	Pericardial Effusion with mass, and fibrin bridges across the pericardial space	AM	Possible Lung by sputum culture & radiologic findings	VOR 200 mg BID PO switched to IV 6 mg/kg BID for 2 days then to 4 mg/kg BID IV + Caspofungin 70 mg D1 & 50 mg daily IV till day 50 then on D90 IV VOR switched to 200 mg BID PO since as 2ry ppx	Alive
(Cooper et al., 1981), CR: 18	33 M	Pleuritic chest pain with high IgE and high eosinophil	Labor in shipyard, peanut & chemical factory, bricklayer apprentice	Flavus	PERI	N.D.	Chest X-ray LUL infiltrate	Cardiac mass on the atrial side of the MV	AM	Lung	ABD (2 g for 2 months then after 5 months 3 g over 2 months then after 8 months another 2 g for 2 months) then ketoconazole for secondary ppx	Alive
(Carrel TP et al., 1991), CR: 19	68 M	Fever post ACB and shock	Triple ACB	Fumigatus	PERI	Serum Galactomannan -ve but +ve in trachea	N.D.	N.D.	BOTH	Lung, pleura, diaphragm PD dialysis fluid	No Treatment	Died within 30 days
(Gorospe et al., 2022), CR: 20	63 F	N.D.	ALL (T-cell) s/p HSCT twice	Fumigatus	PERI	N.D.	RML Nodular necrotic consolidation	Pericardial effusion	AM	Lung	AMB IV, isavuconazole, (Dose/duration N.D.) & 2 weeks later Pericardial drainage	Alive
(Caballero et al., 2016), CR: 21	42 F	Cardiac tamponade on post AVR + MVR op D1 with surgical drains in situ	4 heart surgeries for AVR + MVR	Fumigatus	PERI	Serum Galactomannan -ve but fungitell was +ve	CT Chest negative	N.D.	AM	Sternum	LAmB 3 mg/kg IV daily for 1 week switched to VOR 2.5 mg/kg BID IV for 4 weeks + IV caspofungin of 70 mg D1 then	Alive

											50 mg daily for 3 weeks then back to PO VOR for 3 months	
(Müller et al., 1987), CR: 22	40 M	Progressive SOB, pulsus paradoxus & hypotension	CML S/P BMT	Fumigatus	PERI	N.D.	Lingula, LLL, and right lung infiltrate	Cardiac Tamponade, Pneumopericardium	AM	Possible lungs from radiology findings	AMB (Dose/duration N.D.) then pericardial drain followed by pericardiectomy & pericardial window	Died within 30 days due to pulmonary hemorrhage
(Andersson et al., 1986), CR: 23	48 F	Chest pain, fever & pericardial rub	AML	Species	PERI	N.D.	Signs of fungal pneumonia	N.D.	PM	Lung, pleura, Pulmonary artery and vein, peritoneum with spleen	ABD 0.6 mg/kg/day then switched to encapsulated LAmB (Dose/duration N.D.)	Died (NA)
(Kemdem et al., 2008), CR: 24	19 F	Fever & chest pain	ALL s/p CHOP and allogeneic HSCT	Fumigatus	PERI	Galactomannan +ve in BAL	N.D.	Pericardial effusion and Cardiac mass	AM	Lung & cutaneous nodule	Caspofungin, then voriconazole + pericardial drainage (Dose/duration N.D.)	Died (NA) from cerebral hemorrhage.
(Yu et al., 2020), CR: 25	37 M	Fever, cough, & dyspnea for 2 weeks	Destroyed lung as TB suspected s/p ATT	Fumigatus	PERI	N.D.	Left destroyed + right pleural & pericardial effusion	N.D.	AM	N.D.	Pericardiocentesis then Pericardiectomy & VOR for 3 Month (Dose N.D.)	Alive
(Hori MK et al., 1991), CR: 26	45 M	Wheezing dyspnea cough	Asthma, on steroid, DM	Species	MYO	+ve sputum culture for Aspergillus	Normal	N.D.	PM	Lung and stomach	AMB (Dose/duration N.D.)	Died within 30 days (respiratory failure)
(Ohya et al., 2001), CR: 27	64 M	DIC post transfusion c/b cervical spine hematoma	Cervical disc disease	Species	PAN	It was isolated from lung cystic lesions	N.D.	LLL lesion became cystic	PM	Lung, thyroid, gallbladder, arachnoid	Antimycotic not specified (Dose/duration N.D.)	Died within 90 days

(Navaratnam et al., 2021), CS: 28, 29, & 30	42 F	Tylenol ALP s/p liver transplant	None	Species	MYO	N.D.	Halo sign	Cardiac mass (LV outflow tract)	PM	Lung, Thyroid, Brain, renal	AMB (Dose/duration N.D.)	Died (NA)
	24 M	ALF 2/2 Medications s/p liver transplant.	None	Species	PAN	N.D.	Brain multi Infarct, septic kidneys & spleen emboli	Cardiac mass (RV outflow tract)	PM	Lung & Brain	Fluconazole, AMB, & VOR (Dose/duration N.D.)	Died (NA)
	34 F	Type 1 respiratory failure & seizures	Transplant lung 2/2 CF, hypo-gamma-globulin	Species	MYO	N.D.	Bilateral widespread ground glass changes	MV & AV vegetations	PM	Lung	AMB + Prior VOR & caspofungin treating pulmonary fungal infection. (Dose/duration N.D.)	Died (NA)
(Romagnuolo et al., 2000), CR: 31	37 M	Post liver transplant rash, diarrhea, chest pain	DM, HLP, PBS s/p liver transplant	Species	PAN	N.D.	N.D.	Multiple lung emboli	PM	Lung	AMB (Dose/duration N.D.)	Died within 90 days
(Rouby et al., 1998), CR: 32	75 M	Multiple organ failure	COPD & right heart failure	Species (BAL Fumigatus)	MYO.	Galactomannan -ve in serum but +ve in BAL Cx	Nondilated hypokinetic LV	Plural effusion	PM	Lungs (BAL +ve)	Fluconazole 400 mg BID was given for candidal esophagitis	Died within 30 days
(Sulik-Tyszka et al., 2016), CR: 33	45 M	Fever, SOB, vomit, diarrhea	Mantle cell lymphoma s/p HSCT	Species (BAL Fumigatus)	MYO	Galactomannan +ve.	N.D.	Normal	PM	Lung	LAmB 5 mg/kg/d for 93 days then D/C due to renal side effects	Died after 90 days
(Peterson et al., 1984), CR: 34	36 M	Fever, cough, and fatigue	Myelodysplasia	Nidus	MYO + ENDO	N.D.	Normal	Brain infarct, LUL fungus ball	PM	Lung	ABD for a week then restarted after 2 months for 2 days before patient died	Died within 90 days

(Bhat et al., 2012), CR: 35	63 M	N.D.	Hepatitis B cirrhosis s/p Liver transplant	Flavus	PERI	N.D.	Cardiac tamponade	N.D.	AM	N.D.	Pericardial window then Voriconazole & Caspofungin (Dose/duration N.D.)	Died after 90 days "Acute aspiration pneumonia"
(Rueter et al., 2002) CR: 36	65 M	Fever, cough, & high respiratory rate	Ischemic CMP s/p heart transplant	Species (BAL Fumigatus)	MYO + ENDO	N.D.	Decreased ejection fraction	LUL + RML + LU bronchus masses	PM	Lung and brain	IV LAmB for 7 days (Dose N.D.)	Died after 90 days
(Jones et al., 2019), CR: 37	50 F	Blurred vision and pyrexia	Renal transplant	Fumigatus	ENDO like mass	N.D.	LV & basal septum mass	N.D.	AM	Lung, eye, ankle	Surgical mass excision with recent unspecified invasive pulmonary aspergillosis Tx	Alive
(Lim et al., 1997), CR: 38	39 F	Weakness, SOB, painful right hand skin nodules	Sjogren's, Renal IgA on chronic steroid	Species	ENDO like mass	N.D.	Mass on the papillary muscle of LV	Basal ganglia suspected cerebritis or abscess.	AM	Skin & possibly brain MRI	Cardiac surgical mass excision	N.D.
(Pavlina et al., 2018), CR: 39	70 F	Right upper limb weakness and confusion	B-Cell CLL	Fumigatus	ENDO like mass	Galactomannan +ve in serum	Multiple bilateral rim enhancing brain lesions	Mass on the inferior wall of the LV	AM	Brain	VOR 6 mg/kg Q12h IV on day 1 then 4 mg/kg Q12h IV + Micafungin 100 mg QD IV for 30 days + LV Mass Excision & cardiac muscles debridement	Died within 30 days "post op D17 of Tt & D30 from multiorgan failure"
(Poupelin et al., 2006), CR: 40	56 M	Fever, SOB, hepatitis and glossitis	COPD, Sarcoid of the lung	Fumigatus	PERI	Galactomannan +ve in serum	Mediastinal LAD, diffuse infiltrate, plural + pericardial effusion	Pericardial effusion then cardiac tamponade	AM	Lung	Pericardial drainage, PO VOR 6 mg/kg/d for 2 days, then 4 mg/kg/day + caspofungin 50 mg/d 5 days later for 4 weeks while VOR for 3 months	Alive

(Delcroix G et al., 2006), CR: 41	31 F	Abdominal pain, jaundice, & fever with hemolytic anemia, DIC.	Hemo-phagocytic syndrome (+ve EBV, cmv/hsv, mumps, Parvovirus B19, mycoplasma pneumonia)	Species (BAL Fumigatus)	PERI	Serum PCR +ve Fumigatus	Ascites & HSM. Mycotic esophagitis PET +ve Pericardium + lung foci X2	Cardiac Tamponade	AM	Lung	Fluconazole for esophagitis. For aspergillosis, VOR complicated liver toxicity switched caspofungin (Dose/duration N.D.)	Died (NA) from multiple: viral, mycobacterial and fungal
(WELSH & BUCHNESS, 1955), CR: 42	18 M	Recurrent fever with leukopenia	Splenectomy for primary splenic neutropenia	Flavus (RLL Fumigatus and RUL Nidulans)	MYO + ENDO	N.D.	Diffuse nodules BL, coarse left lung infiltrate	N.D.	PM	Lung	No treatment	Died within 90 days
(Rogers et al., 1990), CR: 43	69 M	Fever, Respiratory failure sp ACB/ AICD	IHD & Arrhythmia	Species (Flavus in lung)	PERI + MYO	N.D.	LLL consolidate and RLL air fluid level	N.D.	PM	Lung	No treatment	Died within 90 days
(Sergi et al., 1996), CR: 44	55 M	Abscess Perianal drainage POD 2 Fever & POD 5 low BP, necrotizing fasciitis pelvis	No known conditions. At autopsy, found liver cirrhosis & splenic CA	Species	PAN	No serum precipitating ABs to Aspergillus Ag spp. were found on POD 7 & 12	Necrotizing fasciitis of the AP wall, retroperitoneum + right thigh	N.D.	PM	Lung, brain, kidney and thyroid gland.	No treatment	Died within 30 days
(Kaplan R et al., 1981), CR: 45	43 M	kidney transplant c/b CPA	HTN, ESRD s/p Kidney transplant	Fumigatus	PAN	N.D.	N.D.	N.D.	PM	Lungs	No treatment (Failed pericardiocentesis)	Died within 30 Days
(Salanitri et al., 2005), CR: 46	33 M	Chest pain, dyspnea	HIV	Species	PERI	N.D.	Abnormal cardiac contour + effusion	Pericardial effusion	AM	Lung	No treatment (Palliative for Dx pseudoaneurysm mycotic sinus)	N.D.

(Lang DM et al., 1988), CR: 47	42 F	Recurrent ulceration of hand and feet	SLE, Raynaud, recurrent ulcers	Fumigatus	PAN	N.D.	Normal X-Ray	Pericardial effusion	AM	Left hand, Right foot 2/2 thrombi "Left ulnar & Right Anterior tibial artery + posterior tibial artery clot"	ABD 0.6 mg/kg/day, 5-FC 150 mg/kg/day, & rifampin 600 mg daily for 2 weeks (D13 – D28) + D23 MV & papillary Muscle excision/exchange (porcine xenograft)	Died within 30 days "D5 postop 2/2 RF & shock 2/2 E/Cloacae Pneumonia"
(Xie et al., 2005), CR: 48	62 M	Fever with decreased breath sounds, holosystolic MV murmur	AIDS, DM, HTN	Species	PAN	N.D.	Bilateral pneumonia & multiple infarct and midline shift	MV veg	PM	Heart, brain, kidneys, & adrenal glands	No treatment	Died "VTach and stroke".
(Kombade et al., 2018), CR: 49	67 M	Chest pain, SOB and cough for one month, & diarrhea	Lung TB On ATT	Nidulans	N.D.	N.D.	Plural & Pericardial effusion + thickening	Pericardial thickening and effusion	AM	N.D.	No treatment (D/C AMA)	Unknown (Discharged AMA and lost follow up)
(Cishek et al., 1996), CR: 50	40 F	Bloody diarrhea, abdominal pain, fever & chest pain.	Ulcerative colitis, tamponade 2/2 pericarditis "bacterial"	Species	PAN	N.D.	N.D.	Pericardial effusion	PM	lungs, kidneys, brain, liver, thyroid, spleen	Pericardiocentesis and pericardial drain placed	Died within 30 days
(Vaideeswar, 2010), CS: 51, and 52	23 M	Fever, chills, hemoptysis, abdominal pain, oliguria. +ve leptospirosis.	Works as a diamond cutter.	Species	PAN	N.D.	ARDS, medical renal disease	N.D.	PM	Aorta, lungs, & brain.	No treatment.	Died within 30 days.

	24 M	Fever, ocular pain, SOB, loose stool, oliguria. +ve leptospirosis.	None	Fumigatus	PAN	N.D.	N.D.	Pericardial effusion and low ejection fraction	PM	Lung, & brain	No treatment	Died within 30 days
(Bullis et al., 2019), CR: 53	60 F	Sob & chest pain of 3 weeks	Churg Strauss syndrome	Fumigatus	MYO	Galactomannan -ve in BAL & BAL	Pulmonary edema, LUL infiltrates, BL effusion	Low ejection fraction 15%	PM	N.D.	No treatment	Died within 30 days
(Chatterjee et al., 2014), CS: 54, and 55	29 M	Fever, SOB, cough and hemoptysis	None	Flavus	PAN	N.D.	Soft tissue mass encases the heart	MV vegetation	PM	Kidneys and spleen	No treatment	Died within 30 days.
	40 M	Fever, SOB, hemoptysis, & hematuria	Smoker	Species (BAL Fumigatus)	PAN	N.D.	Enlarged heart	Pericardial effusion & MV mass	PM	Pancreas, and kidneys	No treatment	Died with 30 days.
(Miyoshi et al., 2006), CR: 56	76 M	Left psoas mass	Malignant lymphoma of psoas muscle	Species	PERI	N.D.	BL pleural effusion. Brain Metastasis	N.D.	PM	Lung, kidney, & pancreas.	Antimycotic not specified (Dose/duration N.D.)	Died at D9 (8 months after psoas mass)
(Cabot et al., 1976), CR: 57	54 M	Post AVR drainage from scar & fever with pericardial rub	DM, dental infection before AVR	Species	PERI + ENDO	N.D.	Heart enlarged after AVR	AV Veg	PM	N.D.	AV replacement	Died within 30 days (After AVR)
(Dimopoulos et al., 2017), CR: 58	79 M	N.D.	None	Species	PERI + ENDO	N.D.	Bilateral pneumonia	N.D.	PM	Lung, kidneys, abdomen, liver	No treatment	Died (NA)
(ST. PIERRE et al., 1998), CR: 59	28 F	SLE	SOB, BLE edema	Species	MYO	N.D.	Low EF 10-15%	N.D.	PM	N.D.	AMB (Dose/duration N.D.)	Death within 30 days

(Arthur H. Williams, 1974), CS: 60, and 61	66 M	Fever, respiratory symptoms, and hypotension	Smoker, suspected TB s/p ATT + Lung CA s/p cobalt Tt	Fumigatus	MYO	N.D.	N.D.	LUL cavity	PM	Lung, renal, thyroid, and brain	No treatment	Died within 30 days.
	60 M	Post Left tibia ORIF Fever, SOB, & AMS	Partial gastrectomy + Halothane liver toxic	Flavus	MYO + ENDO	N.D.	N.D.	Pulmonary embolism	PM	Lung, spleen, kidneys, thyroid, and brain	No treatment	Died within 30 days
(Cox et al., 1990), CR: 62	31 M	Fever, vomit, headache, and holosystolic murmur	HIV with OIs (PJP, Toxoplasma)	Fumigatus	MYO + ENDO	N.D.	Cardiac mass at LV.	Multiple frontal cortex hypodense lesion/edema	PM	Brain, spleen, kidney and pancreas	No treatment (Received Anti toxoplasma & bactrim for PJP)	Died (NA)
(Ross et al., 1985), CR: 63	31 M	Fever, low WBC Counts, pulmonary infiltrate	Lymphoma s/p Chemo + cranial radiation	Fumigatus	PERI + coronary sinus & ostia	N.D. (fungal culture tests were negative)	Normal ECHO	N.D.	PM	Lung	ABD + Rifampin (Dose/Duration N.D.)	Died within 90 days
(van Ede et al., 1994), CR: 64	29 M	Neutropenic fever with precordial pain	AML M3 S/P splenectomy and CHOP at age 14 with new recurrence AML M1	Species (BAL Fumigatus)	PERI	Galactomannan +ve in serum & Aspergillus Fumigatus BAL	Air in the Pericardial space with a slight amount of fluid	Bilateral infiltrate + pneumopericardium	PM (AM BAL +ve but PM myocardial autopsy finding +ve).	Lung	ABD 1mg/kg/d & D/C 1mg/kg 3 X/week + Itraconazole 400 mg BID but readmitted after 3 weeks, continued Itraconazole. A month later had relapse AML s/p CTOP c/b worsening Sx with re-start ABD 1mg/kg/day but died after 48 hours	Died within 90 days due to respiratory failure

(Carrascosa Porras et al., 2002), CR: 65	58 M	Cough & SOB for 2 weeks (Recent +ve sputum Cx for Aspergillus 1 month ago)	COPD + Chr Liver alcoholic disease	Species	MYO	N.D.	N.D.	1st CXR norma then 2nd with minimal LLL opacity + EKG AVNRT	PM	N.D. (Lung, esophagus, gastroduodenal tract, colon, kidneys were free)	No treatment	Died within 30 days
(Gomyo H et al., 2003), CR: 66	50 M	Fever D11 post CTOP then had anterior chest pain and developed respiratory symptoms D26 with signs of effusion and AV node dysfunction	ALL s/p Chemo	Species	PERI. Myo, and AV node	1st Pericardial fluid LA +ve and 2nd DNA PCR +ve.	Pleural effusion, RML infiltrate with enlarged cardiac silhouette	Pericardial Effusion & tamponade	AM	Multi organs including lungs, pleura, liver	Fluconazole 200 mg daily on D11 for fever post CTOP then D26 AMB 0.5 mg/kg/d IV (Plural Aspergillus) switched into itraconazole due to LFT abnormality. Later, pericardial aspergillus Dx after D47 (Pericardiocentesis x2) & started AMB 1-5 mg/kg/d + Intrapericardial AMB 1 mg/kg/day + 5-FC Switched Itraconazole 2/2 AKI (Dose/duration N.D.)	Died within 90 days disseminated aspergillosis.
(S Yamamoto et al., 2005), CR: 67	34 M	Fatigue	ALL s/p CTOP and BMT C/B MI	Species	PERI	*Aspergillus* Ag in the blood was negative	6.7×3 cm mass on the outer RA to RV + LAD occlusion	Cardiac mass from the RA to the RV and 6.0 cm	AM	N.D.	Mass resection & 1-V ACB then Antifungal not specified up till 7 months post-op	Alive

#: Number; **N.D.**: Not done or not documented; **C/B**: Complicated by; **S/P**: Status post; **2/2**: Secondary to; **D/C** or **DC**: Discharge; **AMA**: Against medical advice; **Chr**: Chronic; **F**: Female; **M**: Male; **Pt**: Patient; **Cx**: Culture; **PPx**: Prophylaxis; **Tx**: Treatment; **+ve**: Positive; **-ve**: Negative; **BL**: Bilateral; **CT**: Computed Tomography; **MRI**: Magnetic Resonance Imaging; **AM**: Antemortem; **PM**: Postmortem; **Spp.**: Species; **PERI**: Pericardium; **MYO**: Myocardium; **ENDO**: Endocardium; **PAN**: Pancardium (ENDO + MYO + PERI); **TTE**: Transthoracic Echocardiogram; **TEE**: Transesophageal Echocardiogram; **PTCA**: Percutaneous Transluminal Coronary Angioplasty;

ACB: Aortocoronary Bypass; **CMP**: Cardiomyopathy; **AICD**: Automated Implantable Cardioverter Defibrillator; **CPA**: Cardiopulmonary Arrest; **Vfib**: Ventricular Fibrillation; **Vtach**: Ventricular Tachycardia; **RA**: Right Atrium; **RV**: Right Ventricle; **LA**: Left Atrium; **LV**: Left Ventricle; **AV**: Aortic Valve; **MV**: Mitral Valve; **TV**: Tricuspid Valve; **PV**: Pulmonary Valve; **LAA**: Left Atrial Appendage; **Veg**: Vegetation; **IV**: Intravenous; **PO**: Orally; **mg**: Milligram; **kg**: Kilogram; **POD**: Post-operative Day; **D**: Day; **QD**: Daily; **BID**: Twice Daily; **TID**: Three Times Daily; **LAmB**: Liposomal Amphotericin B; **VOR**: Voriconazole; **5-FC**: Flucytosine; **DIC**: Disseminated Intravascular Coagulation; **AML**: Acute Myeloid Leukemia; **ALL**: Acute Lymphoblastic Leukemia; **CML**: Chronic Myeloid Leukemia; **CLL**: Chronic Lymphocytic Leukemia; **BMT**: Bone Marrow Transplant; **CHOP**: Chemotherapy; **GVHD**: Graft-versus-Host Disease; **RUL**: Right Upper Lobe; **RML**: Right Middle Lobe; **RLL**: Right Lower Lobe; **LUL**: Left Upper Lobe; **LLL**: Left Lower Lobe; **DM**: Diabetes Mellitus; **HTN**: Hypertension; **HLP**: Hyperlipidemia; **HIV/AIDS**: Human Immunodeficiency Virus/Acquired Immunodeficiency Syndrome; **OIs**: Opportunistic Infections; **ALF**: Acute Liver Failure; **PBS**: Primary Biliary Cirrhosis; **AKI**: Acute Kidney Injury; **LAD**: Lymphadenopathy; **ORIF**: Open Reduction and Internal Fixation; **MI**: Myocardial Ischemia; **COPD**: Chronic Obstructive Pulmonary Disease; **TB**: Tuberculosis; **ATT**: Antituberculosis Treatment; **CF**: Cystic Fibrosis; **RF**: Respiratory Failure; **ARDS**: Acute Respiratory Distress Syndrome; **SOB**: Shortness of Breath; **BP**: Blood Pressure; **HR**: Heart Rate; **AMS**: Altered Mental Status; **VRE**: Vancomycin-Resistant Enterococci; **EBV**: Epstein-Barr Virus; **CMV**: Cytomegalovirus; **HSV**: Herpes Simplex Virus.
