# Supplementary material for: A Distinct Clinical Entity of Invasive Cardiac Aspergillosis: Not the Heart Valves This Time
Source: J Fungi (Basel). 2025 Jun 26;11(7):486. doi: 10.3390/jof11070486 (PMC12295503; doi:10.3390/jof11070486)
Supplement: Supplementary file 1 [file jof-11-00486-s001.zip › Supplementary Table S2.pdf]

Table S2. Comprehensive details about the reported treatment strategies.

Treatment Strategy	Alive Cases	Alive (n)	Dead Cases	Dead (n)	Total
**Combination Antifungal Tx + Surg/PROC**	(Kupsky et al., 2016) (VOR+AFG+PCC)<br>(Gorospe et al., 2022) (ABD+ISAV+PCW)<br>(Poupelin et al., 2006) (VOR+CAS+PPD)	3	(Walsh & Bulkley, 1982) (ABD+5FC+PCC)<br>(Bhat et al., 2012) (VOR+CAS+PCW)<br>(Pavlina et al., 2018) (VOR+MFG+mass exc/debridement)<br>(Lang DM et al., 1988) (ABD+5-FC+RIF+MV exc/exch)<br>(Gomyo H et al., 2003) (FLZ to AMB+5-FC to ITZ + PCC & iPC AMB)	5	8
**Combination Antifungal Tx**	(Mortensen et al., 2011) (VOR+CAS)<br>(Caballero et al., 2016) (LAmB to VOR+CAS to VOR)	2	(Navaratnam et al., 2021) (FLZ to VOR+AMB)<br>(Navaratnam et al., 2021) **(**VOR+CAS to AMB)<br>(Ross et al., 1985) (ABD+RIF)<br>(van Ede et al., 1994) (ABD+ITZ)	4	6
**Sequential Antifungal Tx + Surg/PROC**	**Our Case** (LAmB to VOR +PCW)<br>(Le Moing et al., 1998) (ABD to ITZ + pericardiectomy, & pleuropericardial fenestration)<br>(Hayashi et al., 2017) (VOR to AMB to VOR + PCC)	3	(Kemdem et al., 2008) (CAS to VOR + PPD)	1	4
**Sequential Antifungal Tx**	(Cooper et al., 1981) (ABD to suppressive KCZ)	1	(Delcroix G et al., 2006) (FLZ to VOR to CAS)	1	2
**Monotherapy Antifungal Tx + Surg/PROC**	(Alkuwaiti et al., 2019) (VOR + Fibro purulent PERI tissue resection)<br>(Guimaron et al., 2022) (VOR + PCC to pericardiectomy)<br>(Yu et al., 2020) (VOR + PCC then Pericardiectomy)<br>(Jones et al., 2019) (AFT + mass resection)<br>(S Yamamoto et al., 2005) (AFT + Mass excision & 1-V ACB)	5	(Biso et al., 2017) (ABD + PCC with PPD)<br>(Dalhoff et al., 1996) (LAmB + PPD including iPC AMB)<br>(Alam et al., 1998) (AMB + surgical excision of the mass)<br>(Itoh et al., 2006) (AMB + failed PTCA)<br>(Luce et al., 1979) (ABD + PCW)<br>(Müller et al., 1987) (ABD + PPD to pericardiectomy)	6	11
**Monotherapy Antifungal Tx**	-	0	(Walsh & Bulkley, 1982) (ABD), (Walsh & Bulkley, 1982) (ABD), (Schwartz, 1989) (ABD), (Andersson et al., 1986) (AMB "ABD to LAmB"), (Hori MK et al., 1991) (AMB), (Ohya et al., 2001) (AFT), (Navaratnam et al., 2021) (AMB), (Romagnuolo et al., 2000) (AMB), (Rouby et al., 1998) (FLZ), (Sulik-Tyszka et al., 2016) (LAmB**),** (Peterson et al., 1984) (ABD), (Rueter et al., 2002) (LAmB), (Miyoshi et al., 2006) (AFT), (ST. PIERRE et al., 1998) (AMB)	14	14
**Surg/PROC***	-	0	(Cishek et al., 1996) (PCC then PPD)<br>(Cabot et al., 1976) (AV replacement)	2	3
**No treatment****	-	0	(Walsh & Bulkley, 1982), (Carrel TP et al., 1991), (WELSH & BUCHNESS, 1955), (Rogers et al., 1990), (Sergi et al., 1996), (Kaplan R et al., 1981) (failed PCC), (Xie et al., 2005), (Vaideeswar, 2010), (Vaideeswar, 2010), (Bullis et al., 2019, Chatterjee et al., 2014)(Dimopoulos et al., 2017), (Arthur H. Williams, 1974), (Arthur H. Williams, 1974), (Cox et al., 1990) (Carrascosa Porras et al., 2002)	17	19
**Total**		14		49	67

*In Surg/PROC, 1 case (Lim et al., 1997) underwent surgical mass excision but outcome wasn't documented. **In patients who didn't receive treatment, the outcome wasn't documented in case (Salanitri et al., 2005) as he became under palliative care and case (Kombade et al., 2018) as he was discharged against medical advice. Antifungal Therapy = Antifungal Tx, Surgery =

Surg, Procedure = PROC, Voriconazole = VOR, Itraconazole = ITZ, Isavuconazole = ISAV, Fluconazole (FLZ), AMB = Amphotericin B, LAmB = Liposomal Amphotericin B, ABD = Amphotericin B deoxycholate. Caspofungin = CAS, Anidfulafungin (AFG), Micafungin (MFG), 5-fluorocytosine = 5-FC, Rifampin = RIF, Pericardiocentesis = PCC, Pericardial window = PCW, Percutaneous pericardial drain = PPD, Intrapericardial = iPC, AV = Aortic Valve, MV = Mitra valve, excision = exc, exchange = exch, Percutaneous Transluminal Coronary Angioplasty = PTCA
